# 2011年美国临床肿瘤学会年会——晚期非小细胞肺癌内科治疗进展

**DOI:** 10.3779/j.issn.1009-3419.2011.12.08

**Published:** 2011-12-20

**Affiliations:** 1 102200 北京，北京市昌平区医院肿瘤科 Department of Oncology, Beijing Changping District Hospital, 102200 Beijing, China; 2 101149 北京，首都医科大学附属北京胸科医院肿瘤内科 Department of Medical Oncology, Beijing Chest Hospital, Capital Medical University, 101149 Beijing, China

2011年6月3日-7日, 第47届美国临床肿瘤学会年会在美国芝加哥举行, 年会上报告了多项晚期非小细胞肺癌(non-small cell lung cancer, NSCLC)内科治疗方面的临床研究结果, 其中靶向治疗的进展尤为突出。本文将年会上有关晚期NSCLC内科治疗的研究进展报道如下。

## 1 一线治疗进展

纳米紫杉醇是与微小白蛋白结合的紫杉醇, 与传统紫杉醇相比, 纳米紫杉醇可以更好地到达肿瘤微环境, 优先被肿瘤细胞摄入。美国Socinski等^[1]^开展的一项Ⅲ期试验比较了纳米紫杉醇联合卡铂(nab-PC)和紫杉醇联合卡铂(PC)一线治疗晚期NSCLC的疗效及安全性。入组的Ⅲb/Ⅳ期NSCLC患者(ECOG 0/1)随机分为接受nab-PC(卡铂AUC=6, 每3周1次; 纳米紫杉醇100 mg/m^2^, 每周1次, *n*=521)或PC(卡铂AUC=6, 每3周1次; 紫杉醇200 mg/m^2^, 每3周1次, *n*=531)。主要终点为客观缓解率(objective response rate, ORR)。nab-PC组ORR明显高于PC组(33% *vs* 25%, *P*=0.005), 两组无进展生存时间(progression-free survival, PFS)无明显差异。nab-PC耐受性好, 有统计学差异的3级毒副反应中, nab-PC组中性粒细胞减少、神经病变、肌肉痛、关节痛发生率低于PC组, 而血小板减少、贫血发生率则高于PC组。这项大样本的研究达到主要终点, ORR明显提高, 而且对患者生活质量影响较大的神经病变等毒副反应发生率低, 纳米紫杉醇联合铂类一线治疗晚期NSCLC是安全有效的。

S-1是一种口服氟尿嘧啶衍生物, 由替加氟、吉莫斯特和氧嗪酸钾三种成分按照摩尔比1:0.4:1的比例组成, 已广泛用于胃肠道等肿瘤的治疗, 在肺癌方面的研究也有较多报道。日本Hirashima等^[2]^报道了LETS研究(WJTOG3605)中总生存期(overall survival, OS)的更新结果。未化疗过的Ⅲb/Ⅳ期NSCLC患者(ECOG 0-1)随机分为卡铂+S-1组(卡铂AUC=5, d1;S-1 40 mg/m^2^, bid, d1-14, *n*=282), 或者卡铂+紫杉醇组(卡铂AUC=6, d1;紫杉醇200 mg/m^2^, d1, *n*=282), 21天为1周期, OS为主要终点。全部患者中, 卡铂+S-1组的中位生存期(median survival time, MST)为15.2个月, 卡铂+紫杉醇组为13.1个月。鳞癌患者中, 卡铂+S-1组MST为14.0个月, 卡铂+紫杉醇组为10.55个月, 这些生存数据重复了既往研究结果, 证实卡铂联合S-1可用于晚期NSCLC一线治疗, 今后有必要开展前瞻性研究评价其在鳞癌患者中的疗效。目前, 我国正进行S-1联合顺铂一线治疗晚期NSCLC的多中心临床试验。

在有*EGFR*突变的高加索患者中比较厄洛替尼与化疗疗效及安全性的研究较少。EURTAC是在欧洲开展的一项Ⅲ期随机研究^[3]^, 比较二者在晚期NSCLC患者中的差异。从2007年2月-2011年1月, 对1, 227例患者进行了*EGFR*突变检测, 有突变的174例患者被随机分至厄洛替尼组或含铂化疗组, 主要终点为无进展生存时间PFS, 次要终点为缓解率(response rate, RR)、OS和毒副反应。年会上报告了153例患者(化疗组76例, 厄洛替尼组77例)的中期结果。化疗组:男性16例, 中位年龄64岁, 从不吸烟者56例, 腺癌67例。厄洛替尼组:男性25例, 中位年龄65岁, 从不吸烟者54例, 腺癌73例。化疗组和厄洛替尼组有效率分别为10.5%、54.5%(*P* < 0.000, 1), PFS分别为5.2个月、9.4个月(*P* < 0.000, 1), OS分别为18.8个月、22.9个月(*P* < 0.42)。常见的毒副反应:化疗组为乏力(68.9%)、贫血(45.9%)、恶心(40.5%)和中性粒细胞减少(36.5%)。厄洛替尼治疗组为腹泻(57.3%), 乏力(53.3%)和皮疹(49.3%)。总生存结果还有待继续随访。研究证实, 在一线治疗有EGFR突变的高加索晚期NSCLC患者上, 厄洛替尼较含铂化疗明显提高了PFS, 毒副反应也可接受。

年会上公布了日本NEJ002研究^[4]^的最终OS结果, 即比较吉非替尼与卡铂联合紫杉醇(CP)一线治疗有*EGFR*突变的晚期NSCLC的研究。吉非替尼组MST和2年生存率分别为27.7个月和57.9%, CP组则分别为26.6个月和53.7%(*P*=0.483), 各亚组OS无明显差异。在吉非替尼组, 19外显子缺失和L858R突变的患者OS相似(28.9个月 *vs* 28.0个月)。二线治疗中吉非替尼组90%患者接受化疗, CP组96%接受吉非替尼治疗。两组OS无明显差异可能与CP组高度交叉使用吉非替尼有关。

OPTIMAL是比较厄洛替尼与吉西他滨+卡铂一线治疗有*EGFR*突变的晚期NSCLC患者的随机开放Ⅲ期研究^[5]^, 结果已证实厄洛替尼组的PFS、ORR和耐受性更佳。本次ASCO更新了OPTIMAL研究的PFS数据, 并首次报道QOL结果。截止到2011年1月7日, 更新的PFS为13.7个月 *vs* 4.6个月(*P* < 0.000, 1)。与化疗组相比, 厄洛替尼组的临床相关QOL也有明显改善。

IPASS等研究结果使晚期NSCLC的一线治疗模式发生了很大的变化, 2011年版NCCN指南推荐EGFR-TKIs作为*EGFR*基因敏感突变的晚期NSCLC一线标准治疗, 对于无敏感突变或突变状况未知患者仍推荐化疗。年会上报告的EURTAC、NEJ002、OPTIMAL三项在高加索患者、亚裔患者中进行的大样本研究, 均支持IPASS的结果。这些研究把靶向治疗推向了一个高潮, 晚期NSCLC患者的治疗已从经验性治疗转向个体化治疗, EGFR-TKIs是*EGFR*突变患者一线治疗的最佳选择。

## 2 维持治疗

PARAMOUNT是探索培美曲塞维持治疗能否延长晚期非鳞NSCLC患者PFS的Ⅲ期临床试验^[6]^。939例未经过化疗的Ⅲb/Ⅳ期非鳞NSCLC一线采用培美曲塞+顺铂化疗4周期, 未进展的患者2:1随机分为培美曲塞维持治疗组(500 mg/m^2^, d1, 21天1周期, *n*=359)或安慰剂维持治疗组(*n*=180), 直至疾病进展, 主要终点为PFS。结果:从随机开始, 培美曲塞组和安慰剂组的PFS分别为3.9个月、2.6个月(*P*=0.000, 2), 疾病控制率(disease control rate, DCR)分别为71.8%、59.6%(*P*=0.009)。PARAMOUNT研究证实培美曲塞作为非鳞NSCLC的维持治疗能延长患者的PFS, 维持治疗有效, 耐受性好, OS结果还在观察中。这是继JMEN试验之后的又一项大样本的Ⅲ期研究。JMEN研究中共663例接受含铂化疗且疾病无进展的晚期NSCLC患者随机接受最佳支持治疗联合或不联合培美曲塞进行维持治疗。接受培美曲塞的患者的无病生存期延长(4.3个月 *vs* 2.6个月, *P* < 0.000, 1);培美曲塞组的OS长达13.4个月, 明显优于安慰剂组10.6个月, 死亡风险明显下降21%(OS, HR=0.79);在非鳞癌患者中, 培美曲塞使总生存期延长(15.5个月 *vs* 10.3个月, *P*=0.002), 而且耐受性良好。PARAMOUN再次验证低毒药物培美曲塞在维持治疗中的作用。

年会上报告的另一项重要的维持治疗研究是INFORM试验^[7]^, 即探索吉非替尼在局部晚期/转移NSCLC患者的维持治疗的疗效和安全性的Ⅲ期多中心随机研究。296例Ⅲb/Ⅳ期NSCLC完成4周期含铂两药化疗后未进展的患者1:1随机为吉非替尼组(250 mg/d)或安慰剂组, 各148例。主要终点为PFS, 次要终点为OS、ORR、DCR、症状改善情况及耐受性。结果:吉非替尼组与安慰剂组PFS分别为4.8个月、2.6个月(*P* < 0.000, 1), ORR分别为23.6%、0.7%(*P*=0.000, 1), DCR分别为71.6%、50.7%(*P*=0.000, 1)。吉非替尼组与安慰剂组严重不良事件发生率为6.8%与3.4%。79例患者完成*EGFR*基因检测, 30例突变阳性(38%)。突变阳性患者中, 接受吉非替尼和安慰剂治疗的PFS分别为16.6个月、2.8个月, 突变阴性PFS分别为2.7个月、1.5个月, 突变状态未知患者的PFS分别为6.0个月、2.7个月。吉非替尼组与安慰剂组腺癌患者的PFS分别为8.5个月、2.6个月。此项研究证实, 吉非替尼维持治疗明显延长了亚裔晚期NSCLC患者的PFS, 并明显提高患者的ORR和DCR, 也延长*EGFR*突变患者的PFS, 耐受性好。

INFORM是首个在亚裔晚期NSCLC患者中开展的随机对照的维持治疗研究, 研究达到主要终点, 证明吉非替尼维持治疗晚期NSCLC患者较安慰剂明显延长无进展生存期, 突变患者和腺癌患者获益更明显, ORR、DCR与PFS结果一致, 吉非替尼维持治疗耐受性良好。

## 3 二线治疗

埃克替尼是一种强效、高特异性的表皮生长因子受体酪氨酸激酶抑制剂。中国研究者报告了一项埃克替尼与吉非替尼的非劣效随机双盲Ⅲ期试验^[8]^结果。共399例一线或二线化疗后进展的NSCLC患者被随机分为埃克替尼组(150 mg, tid)或吉非替尼组(250 mg, qd)。主要终点为PFS。埃克替尼组PFS较吉非替尼长35天(137天 *vs* 102天, HR=0.84, 95%CI:0.67-1.05), 达到了非劣效的主要终点, 两组OS无明显差别(504天 *vs* 531天)。埃克替尼和吉非替尼组的ORR (27.6% *vs* 27.2%)、DCR (75.4% *vs* 74.9%)、疾病进展时间(time to progression, TTP)(156天 *vs* 111天)及QOL(101.4±9.6 *vs* 103.0±19.1)也无明显差别。埃克替尼组毒副反应发生率明显低于吉非替尼组(60.5% *vs* 70.4%, *P*=0.04), 皮疹发生率为39.5% *vs* 49.2%, 腹泻为18.5% *vs* 27.6%, 转氨酶增高为8.0% *vs* 12.6%。进行*EGFR*基因突变分析的132例患者中66例有突变(50%), 其中埃克替尼组27例(40.9%), 吉非替尼组39例(59.1%), 两组中基因突变(M)患者和野生型基因(W)患者的ORR和PFS有明显差别。埃克替尼组中, M *vs* W的ORR为59.3%(16/27) *vs* 5.1%(2/39), PFS为198天 *vs* 70天。吉非替尼组中, M *vs* W的ORR为52.6%(20/39) *vs* 3.7%(1/27), PFS为158天 *vs* 76天。这项试验是埃克替尼与吉非替尼的头对头比较, 结果显示二者疗效相当, 埃克替尼在安全性方面更有优势。基于这个研究结果, 2011年6月中国SFDA批准埃克替尼上市, 用于治疗既往接受过至少一个化疗方案失败后的局部晚期或转移性NSCLC。

韩国Ahn等^[9]^设计的一项随机Ⅲ期试验, 比较吉非替尼和培美曲塞二线治疗晚期肺腺癌患者的疗效。入组患者从不吸烟, 且既往接受含铂化疗, *EGFR*有突变的患者不包括在内。135例患者随机分为接受吉非替尼(250 mg, 口服, qd)或培美曲塞(500 mg/m^2^, d1, 21天为1周期)治疗。主要终点PFS。吉非替尼组和培美曲塞组ORR分别为30.1%和14.9%(*P* < 0.001), PFS分别为9.4个月和2.9个月(*P*=0.010), 研究达到主要终点, 两组均未达到OS。吉非替尼组和培美曲塞组1年生存率分别为73.6%和70.5%(*P*=0.89)。研究显示, 对于临床上经选择的韩国NSCLC患者的二线治疗, 吉非替尼疗效优于培美曲塞。

Ardizzoni等^[10]^通过对两个随机Ⅱ期试验(GOIRC 02.2006和NVALT-7)结果进行合并分析, 评价培美曲塞联合卡铂二线治疗晚期NSCLC能否提高疗效。共479例患者被随机分配接受培美曲塞(500 mg/m^2^), 或培美曲塞联合卡铂(AUC=5, 3周为1周期)治疗。全部患者中, 联合卡铂组总有效率增加, PFS无明显延长, 并未提高生存率。亚组分析中, 接受联合化疗的鳞癌患者PFS明显提高, 从2个月提高至3.2个月, OS从5.4个月提高至9个月。结论:单药培美曲塞方案仍是非鳞肺癌二线治疗的标准方案。尽管培美曲塞无治疗鳞癌的适应症, 但本分析结果支持将来可采用卡铂联合培美曲塞方案二线治疗这种组织亚型的研究。

多西紫杉醇、培美曲塞、厄洛替尼、吉非替尼是目前晚期NSCLC的二线治疗标准, 埃克替尼的出现使二线治疗又有了一个新的药物, 且埃克替尼的毒副作用更轻。随着埃克替尼的上市, 其在晚期NSCLC患者中的作用将得到充分的验证。

## 4 老年患者的治疗

为评价多西他赛联合铂类能否提高疗效, 日本在老年NSCLC患者中开展了一项Ⅲ期随机试验^[11]^, 比较多西他赛(D)与顺铂(P)联合方案周疗与D单药三周方案治疗的疗效(JCOG0803/WJOG4307L组内试验)。未接受过化疗、Ⅲ/Ⅳ期或复发NSCLC、≥70岁、PS 0-1的患者随机分为DP组或D组。DP组:多西他赛20 mg/m^2^+顺铂25 mg/m^2^, d1, d8, d15, 4周为1周期, D组:多西他赛60 mg/m^2^, d1, 3周为1周期。主要终点为OS。276例患者随机分组, DP组和D组的MST分别为13.3个月、17.3个月[HR (95%CI):1.557 (0.976-2.485)]。D组患者生活质量评分占优。结论:顺铂与多西他赛联合周疗方案在老年晚期NSCLC一线治疗上并不优于单药多西他赛。

Guetz等^[12]^对单药化疗与含铂或不含铂两药化疗治疗老年晚期NSCLC患者的疗效和安全性进行了*meta*分析。纳入12项研究, 包括2, 530例患者(中位年龄74岁; 男性1, 911例, 女性545例; Ⅲb期591例, Ⅳ期1, 600例; 鳞癌796例, 腺癌860例)。与单药化疗比较, 两药化疗组的1年生存率得到明显改善(HR=0.92;95%CI:0.87-0.99), ORR的危险比为1.40(95%CI:1.21-1.63;*P*=0.004), 含铂两药化疗也明显地提高OS(HR=0.82;95%CI:0.73-0.93)。两药化疗组的恶心/呕吐、中性粒细胞减少、血小板减少和贫血的风险比明显高于单药组(*P*=0.001)。3/4级毒副反应中, 两药化疗组仅中性粒细胞减少, 血小板减少和贫血的风险明显高于单药化疗组。这项*meta*分析显示, 与单药化疗相比, 两药化疗可以明显提高老年晚期NSCLC患者的ORR和OS, 特别是在和铂联合化疗时, 但血液学毒副反应也更高。

美国肺癌患者的中位年龄是70岁, 我国老年肺癌患者的比例也在增高。单药化疗是老年晚期肺癌患者的标准治疗, 但也有研究证实, PS评分好的老年患者可接受含铂两药方案化疗。鉴于老年患者独特的器官功能特点, 临床上更要注重个体化治疗。

## 5 其它药物

T790M位点突变是一种最常见的TKI获得性耐药机制。阿法替尼(BIBW 2992)(A)是一种不可逆的erbB家族阻滞剂, 在临床前试验中表现出抗T790M活性。Yamamoto等^[13]^在一项Ⅱ期试验中采用阿法替尼治疗既往接受过厄洛替尼(E)或吉非替尼(G)的NSCLC患者。入组标准:Ⅲb/Ⅳ期、ECOG PS 0-1, 经过一二线化疗和≥12周的E或G治疗出现疾病进展的日本NSCLC患者, 接受A 50 mg, 口服, qd。主要终点为ORR。62例患者(女性48例, 男性14例), 11.3%、79%和9.7%分别接受过E、G或两者治疗, 65%患者经过E/G治疗后有缓解。E/G治疗结束到开始A治疗的中位时间为3周。73%患者的原发性肿瘤有*EGFR*突变阳性。82%患者符合E/G获得性耐药的定义:①*EGFR*突变阳性或②E/G治疗后完全缓解(complete response, CR)/部分缓解(partial response, PR)或③E/G治疗后疾病稳定(stable disease, SD)至少6个月和④E/G治疗结束后至少4周。结果:PR为13%, DCR(> 8周)为72%, 获得性耐药患者PR为5.9%, DCR为69%, PFS为4.4个月。2例患者的原发性肿瘤有T790M突变, 其中1例为L858R+T790M突变患者疾病稳定长达308天。常见的毒副反应为腹泻、皮疹、口腔炎、指甲异常和食欲降低。本试验大多数患者符合NSCLC第一代EGFR-TKIs获得性耐药标准, 试验证实阿法替尼可使这些患者临床获益(包括 > 8周DCR为69%、PFS为4.4个月)。

有研究显示, 阿法替尼和西妥昔单抗联合治疗T790M转基因鼠模型几乎得到了完全缓解。美国Janjigian等^[14]^实施了一项阿法替尼联合西妥昔单抗治疗厄洛替尼或吉非替尼获得性耐药NSCLC的研究, 评价其安全性和初步疗效。临床定义的获得性耐药NSCLC患者口服阿法替尼(40 mg, qd)和剂量逐渐增加的西妥昔单抗(每周2次, 250 mg/m^2^和500 mg/m^2^)。对接受推荐Ⅱ期剂量(RP2D)的患者进行客观缓解的评价。26例临床获得性耐药患者中的22例接受了预先定义的最大剂量(RP2D:阿法替尼40 mg和西妥昔单抗500 mg/m^2^)。未观察到剂量限制性毒性。常见的毒副反应为:1/2级皮疹(35%/46%)和腹泻(50%/19%)、3例(11.5%)发生3级皮疹。所有接受RP2D的患者均表现为疾病控制状态(肿瘤缩小至76%, 治疗时间 > 5个月)。可评价患者中, 8/22(36%)达到PR, 其中4/13(29%)的PR发生在T790M突变患者中。结论:患者对推荐的Ⅱ期剂量的阿法替尼联合西妥昔单抗是可耐受的。由于此方案对既往厄洛替尼或吉非替尼产生获得性耐药患者的临床疗效令人鼓舞, 所以本试验在进一步扩大中。

ALK是肺癌最新的一种酪氨酸激酶靶点。Ⅰ期试验中, ALK酪氨酸激酶抑制剂克唑替尼在ALK阳性NSCLC表现出明显的抗肿瘤活性。但缺少随机的试验数据, 克唑替尼对ALK(+) NSCLC OS的影响尚不明确。Shaw等^[15]^以既往研究为对照, 评价克唑替尼对晚期ALK阳性NSCLC患者生存的影响。研究者观察Ⅰ期试验中82例接受克唑替尼治疗的ALK(+)患者的生存情况(这项研究由Kwak等于2010年发表在新英格兰医学杂志), Ⅰ期试验中37例未接受克唑替尼治疗的ALK(+)患者为ALK(+)对照组, 另外253例ALK(-)/EGFR(-)患者为ALK(-)对照组。82例克唑替尼治疗的ALK(+)患者的1年生存率为77%, 2年生存率为64%, 中位OS尚未达到。37例ALK(+)对照组的1年和2年的生存率分别为73%、33%, MST为20个月。32例ALK(+)患者二/三线接受克唑替尼治疗的生存明显优于ALK(+)对照组, 1年生存率分别为71%、46%(*P* < 0.004), 2年生存率为61%、9%, 治疗患者的中位生存尚未达到, 对照组OS为11个月。123例ALK(-)对照患者的二线治疗的1年、2年生存率为49%、33%, MST为11个月。结论:虽然这不是一项直接进行的比较试验, 但与未接受治疗的对照比较, 克唑替尼能明显延长ALK(+) NSCLC的生存期, 克唑替尼可成为ALK(+) NSCLC患者治疗的一个新标准。

意大利CrinoL等^[16]^报道了克唑替尼治疗晚期ALK阳性NSCLC患者的Ⅱ期试验的初期结果(PROFILE 1005)。该试验包括12个国家57个地区的ALK阳性的复发/晚期/转移的NSCLC患者, 曾接受过≥1次的化疗。患者口服克唑替尼250 mg, bid, 连续3周为1周期。结果:到目前为止, 136例患者可作安全性分析, 76例患者可评价疗效。76例患者中的63例(83%)肿瘤有缩小(41例PR, 54%), 7例病情进展。与治疗相关的常见毒副反应为:恶心(46%)、视觉障碍(45%)、呕吐(39%)和腹泻(29%), 一般为1/2级。3/4级毒副反应发生率为15%(主要为谷丙转氨酶升高、呼吸困难和中性粒细胞减少)。9例死亡患者中有2例与治疗相关(肺炎及不明原因)。大多数患者在治疗的第2个周期时, 临床上疼痛、咳嗽、呼吸困难和乏力有明显改善, 只有便秘发生率在治疗结束时有所增加。这项全球第二大临床研究显示, 克唑替尼安全, 耐受性好, 初期结果证实克唑替尼在既往治疗过的ALK阳性NSCLC患者中, 可缓解症状, 并表现出很好的临床抗肿瘤活性。

基于几项临床研究结果, 2011年8月26日美国FDA批准克唑替尼上市, 用于ALK阳性的晚期NSCLC的治疗。尽管仅7%的NSCLC表达*ALK*基因, 接受克唑替尼治疗, 但在美国就会有10, 000例患者能获益。

*Met*基因表达与多种肿瘤预后差相关, 其中包括NSCLC(NSCLC患者Met突变占8%), Met活化也是EGFR-TKI耐药原因之一。MetMAb是一个单克隆抗体, 能够专门阻断Met受体。年会上报告了OAM4558g研究^[17]^的最终疗效, 这是一项全球范围进行的比较MetMAb+厄洛替尼(ME)与厄洛替尼+安慰剂(PE)二/三线治疗NSCLC的Ⅱ期随机双盲研究。收集肿瘤标本评估c-Met IHC表达水平。主要终点为Met(+)患者的PFS及全部患者的PFS。128例NSCLC患者被随机分为接受ME或PE治疗。中位随访时间为9.9个月。在Met(+)组中, ME组的PFS和OS均有明显提高。全部人群中, PE组和ME组的MST分别为8.2个月和7.1个月(*P*=0.76), c-Met IHC(+)的MST分别为4.6个月和12.6个月(HR=0.37, *P*=0.002), FISH(-)/IHC(+)的MST分别为3.6个月和7.1个月(*P*=0.09), c-Met IHC(-)的MST分别为9.2个月和5.5个月(*P*=0.021), MET FISH(+)NSCLC和FISH(-)/IHC(+)患者的OS均有提高。两组与厄洛替尼相关的毒副反应相似。结论:ME明显提高Met高表达患者的PFS和OS, 死亡风险降低3倍。在FISH(-)/IHC(+)患者中, 观察到IHC可能是判断MetMAb获益更敏感的预测指标。

Yu等^[18]^在年会上对OAM4558g研究中的探索性生物标志进行了分析。通过FISH、qRT-PCR方法或各种突变检测技术对有记录的肿瘤标本进行与c-Met和/或EGFR信号相关的肿瘤生物标志物检测。结果显示:Met、HGF、EGFR、AREG或EREG mRNA高表达水平(≥中位值)不是OS获益的独立预测因素。试验数据支持c-Met IHC是预测MetMAb延长OS的敏感标志物和独立因素, 生物标志物的作用值得进一步探索。

Vashishtha等^[19]^报告了OAM4558g研究中各亚组的毒副反应。结果:除了ME组外周性水肿发生率略高外, MetMAb+厄洛替尼(ME)组与厄洛替尼+安慰剂组(PE)毒副反应相似, ME联合方案基本上没有改变厄洛替尼的安全性范围。

## 6 小结

ASCO是全球范围内肿瘤临床研究的最重要的会议之一, 2011年年会展现了晚期NSCLC内科治疗的多项试验结果, 尤其在维持治疗、二线治疗及一些新的靶向药物的研究等是今年年会的亮点。

多项试验证实, 基于分子标志物的靶向治疗已经开启了NSCLC患者治疗的新领域。今后, 如何通过转化性研究, 不断地发现预测疗效的生物标志、发现肺癌的驱动基因, 从而使肺癌的治疗更加个体化、更加精准, 为患者带来更多的益处将是肺癌研究的重要方向。
